# The Role of Controlled Surface Topography and Chemistry on Mouse Embryonic Stem Cell Attachment, Growth and Self-Renewal

**DOI:** 10.3390/ma10091081

**Published:** 2017-09-14

**Authors:** Melanie Macgregor, Rachel Williams, Joni Downes, Akash Bachhuka, Krasimir Vasilev

**Affiliations:** 1 School of Engineering, Future Industries Institute, University of South Australia, Mawson Lakes, SA 5095, Australia; melanie.macgregor@unisa.edu.au; 2 Institute of Ageing and Chronic Disease, University of Liverpool, Liverpool L7 8TX, UK; jemd@liverpool.ac.uk; 3 Institute for Photonics and Advanced Sensing, University of Adelaide, Adelaide, SA 5000, Australia; akashbachhuka01@adelaide.edu.au

**Keywords:** plasma polymer, Polyallylamine, polyoctadiene, polyacrylic acid, nanotopography, Mouse embryonic stem cells, fibronectin, Extra Cellular Matrix physical cues, Extra Cellular Matrix chemical cues

## Abstract

The success of stem cell therapies relies heavily on our ability to control their fate in vitro during expansion to ensure an appropriate supply. The biophysical properties of the cell culture environment have been recognised as a potent stimuli influencing cellular behaviour. In this work we used advanced plasma-based techniques to generate model culture substrates with controlled nanotopographical features of 16 nm, 38 nm and 68 nm in magnitude, and three differently tailored surface chemical functionalities. The effect of these two surface properties on the adhesion, spreading, and self-renewal of mouse embryonic stem cells (mESCs) were assessed. The results demonstrated that physical and chemical cues influenced the behaviour of these stem cells in in vitro culture in different ways. The size of the nanotopographical features impacted on the cell adhesion, spreading and proliferation, while the chemistry influenced the cell self-renewal and differentiation.

## 1. Introduction

Stem cell-based approaches are one of the greatest areas of promise for modern tissue engineering and regenerative medicine [1,2,3]. In fact, owing to their unique dynamic behaviour, defined as their ability to sense and respond to their environment, the therapeutic prospects of stem cell therapies even expand to the fields of cancer cure [4], diabetes [5], and autoimmune disease [6] treatment. In all cases, following isolation from the native microenvironment, the success of stem cell research and therapy depends on the effective in vitro propagation of the cells before they are eventually transplanted into the host. However, to this day, stem cell-based therapeutic strategies are often met with inconsistent results due to a lack of appropriate understanding of cell-niche interactions [7]. It is well established that, more than any other type of cells, the fate of stem cells, including adhesion, growth, differentiation and apoptosis, is heavily dependent on the cell’s interaction with their physical, mechanical and biological microenvironment. However, the role of physicochemical material properties in modulating intracellular signalling is often overlooked [8]. This is despite increasing evidence acquired both in vivo and in vitro suggesting that, beyond soluble factors and cell-cell interaction, the surface properties of the cellular niche act as potent stimuli in the regulation of biological processes [9]. For instance, it is well accepted that the chemistry and topography [10] of the culture substrate play an important role in directing cell adhesion and spreading, and eventually also their differentiation pathways [9,11,12,13,14,15]. A culture material may be chosen for its ability to maintain cells in an undifferentiated state for as long as possible (for cell therapy), or to direct their differentiation into a targeted cell type (for tissue engineering) [16]. In both cases, the success of stem cell culture relies on understanding which factors influence the stem cell response, and our ability to control them. Yet, establishing model culture materials in which we can control chemistry and nanotopography independently is a challenge [17]. 

Here, we used plasma-assisted surface modification as a novel approach to control surface properties with the potential to influence the fate of the stem cells [18,19]. Using plasmas produced from volatile organic precursors to form thin polymeric films on surfaces, plasma polymer deposition is a highly reproducible and environmentally friendly, one-step coating technique employed to modify the chemistry, wettability and topography of biomaterials [20,21,22,23,24]. Furthermore plasma deposition has added benefits since the process is not dependent on the substrate material, which is an advantage over other methods such as layer-by-layer deposition and SAMs which require specific surface pre-modification [25,26]. The plasma polymers studied used precursors of allylamine, acrylic acid, and octadiene, to produce surfaces with amine, carboxylic acid, and methyl functionalities respectively. The charged nature of the amine and carboxylic acid modified surfaces are known to influence protein adsorption and subsequent cellular interactions with surfaces [12], and the non-polar methyl functional groups are a useful comparison. Tailored nanotopographic features can be created on a surface through the attachment of gold nanoparticles [27]. In this study we created substrates with tailored nanotopography and chemistry and used these to study the response of murine embryonic stem (mES) cells in terms of adhesion, spreading, proliferation, and differentiation. We used the E14 mES cell line, which are blastocyst-derived, owing to its reported controlled behaviour in similar studies [9,15]. Specifically, tailoring the adhesivity of E14 cells by modification of the substrate surface chemistry was shown to restrict their spreading and helped to maintain their capacity to self-renew. Our systematic study decoupled the individual effects of topography and chemistry and revealed that, while topography played a role in cell adhesion, spreading and growth, only chemistry affected stem cell differentiation. More precisely, surfaces with 16 nm nanotopography hindered mES cell growth, and surface chemical functionalisation with polyacrylic acid reduced the degree of pluripotency retention.

## 2. Materials and Methods 

### 2.1. Substrates

Preparation of the model cell culture substrate consisted of four distinct steps: (1) gold nanoparticle synthesis; (2) allylamine plasma polymer coating; (3) surface immobilisation of the nanoparticles; and (4) surface overcoating with selected plasma polymer film. Through this process we created 16 different substrates: nanotopographies of smooth (control), 16 nm, 38 nm, and 68 nm, and for each the surface chemistry of untreated, allylamine, acrylic acid, and octadiene.

### 2.2. Plasma Polymer Coating

Glass coverslips were coated with organic thin films by plasma deposition of allylamine in a custom-made, capacitively coupled plasma reactor as described elsewhere [26,28]. Briefly, a glass chamber comprising two brass electrode set 10 cm apart was brought to a base pressure of 2 × 10^−2^ mbar. Clean glass coverslips were introduced into the vacuum chamber and primed with air plasma for 2 min at 50 W. The allylamine precursor vapour was then introduced into the chamber through a needle valve until a constant flow rate of 10 sccm was reached. The continuous wave plasma was ignited with 40 W Radio Frequency power and maintained for 2 min. The substrates were kept in petri dishes until further use.

### 2.3. Nanotopography Substrate Modification

In order to generate substrates with nanoprotrusions 16, 38, and 68 nm in size, COOH-functionalised gold nanoparticles were bound electrostatically to substrates coated with allylamine plasma polymer. The gold nanoparticles were synthesised by citrate reduction of hydrogen tetrachloroaurate (HAuCl_4_), and their surfaces were functionalised using 2-mercaprosuccinic acid following established procedures [29]. AA-coated coverslips were immersed in AuNPs solution for 6 h before rinsing off any loosely bound particles with MilliQ water [27]. In order to control the surface chemistry of the nanorough substrate, a thin (<10 nm) plasma polymer layer was thereafter deposited on top of the gold nanoparticle as described above using allylamine (ppAA), acrylic acid (ppAAc), or octadiene (ppOD) precursors. 

Surface nanotopography was confirmed via atomic force microscopy (AFM) measurement, while the surface atomic composition was determined via X-ray photoelectron spectroscopy (XPS), as described previously [30,31]. Briefly, AFM analysis was performed on a NT-MDT NTEGRA Scanning Probe Microscope (Moscow, Russia) atomic force microscope in tapping mode. A gold-coated silicon nitride cantilever with resonance frequency between 50 and 150 kHz was used. Images of 5 μm × 5 μm areas were taken with an amplitude oscillation of 10 nm at a scan rate of 0.5 Hz. The atomic composition was determined using a Spec SAGE XPS (Zurich, Switzerland) equipped with a monochromatic Mg radiation source operating at 10 kV and 20 mA. Survey spectra were recorded over the binding energy range of 0–1000 eV at a pass energy of 100 eV and with 0.5 eV resolution. All spectra were referenced to the C1s neutral carbon peak at 285 eV, to compensate for the effect of surface charging. Casa XPS software was used for the processing and curve fitting of all data. Contact angles were measured in a sessile drop configuration using an OCA, SCA20 Dataphysics instrument (Filderstadt, Germany) and software [32,33].

### 2.4. Cell Culture

Mouse ES cell line E14 (a gift from Mark Boyd, University of Liverpool) were cultured in feeder cell-free conditions following previous protocols [9,15] at passage 24. Cell culture media was advanced Dulbecco’s modified Eagle’s medium (DMEM, Gibco, Paisley, UK) supplemented with 2% Embryonic Stem Cell-grade Fetal Bovine Serum (Sigma, Poole, UK), 1% (*v/v*) l-glutamine, 1% (*v/v*) non-essential amino acids, 0.01% (*v/v*) β-mercaptoethanol (Gibco, Paisley, UK) and 1000 U/mL Leukemia Inhibitory Factor (LIF). Cells were subcultured by trypsinisation every 2–3 days.

### 2.5. Mouse ES Cells Culture on Substrates

Nanoparticle coated and chemically defined cover slips were sterilised in an ultra-violet (UV) cross-linker (Ultra-Violet Products Ltd., Cambridge, UK) at a distance of 10 cm for 5 min prior to use. 20,000 cells per cm^2^ were seeded onto substrates pre-coated with 1 μg mL^−1^ fibronectin in phosphate-buffered saline (PBS) for 40 min. Substrates with cells were cultured in duplicate for 2 h, 24 h or 72 h. 

### 2.6. Immunostaining

Cells were fixed for 5 min in a 4% (*w*/*v*) solution of paraformaldehyde (PFA) in PBS, then permeabilised in 0.1% Triton X100 in PBS for 5 min, and washed twice in PBS. For morphological analysis, cells were incubated in 5 U mL^−1^ phalloidin diluted in PBS containing 1% BSA for 20 min at room temperature. Cells were washed three times in PBS and incubated with 300 nM DAPI (4′,6-diamidino-2-phenylindole) in PBS for 10 min at room temperature. Cells were washed three times in PBS and stored in PBS containing 0.1% PFA. 

For analysis of pluripotency, cells were incubated at room temperature with primary antibody in 1% (*w/v*) BSA in PBS for 2 h. Primary antibodies were used at working concentrations of 20 μg^−1^ for octamer-binding transcription factor 4 (Oct4) (insight Biotech Ltd., Wembley, UK) and 15 μg mL^−1^ for Nanog (R&D Systems, Abingdon, UK). Cells were washed three times in PBS and then incubated for 1 h with 4 μg mL^−1^ of the relevant secondary antibodies (AlexaFluor488 or AlexaFluor594-conjugated). Cells were washed in PBS and incubated with 300 nM DAPI in PBS for 10 min at room temperature. Cells were washed three times in PBS and stored in PBS containing 0.1% PFA. Staining was analysed on a Nikon Eclipse TiE at a magnification of 20×.

### 2.7. Cell Adhesion, Morphology and Pluripotency

Attached cells were counted at 2 h, 24 h, or 72 h in eight random fields of view across the duplicate samples under phase contrast microscopy prior to fixing and staining. Mean cell area was calculated using Image J software analysis of images from microscopy of phalloidin stained cells from five random fields of view from one set of the duplicate samples. Percentage pluripotency was calculated using Image J software to assess ratios of DAPI and Oct/Nanog-stained nuclei from three random fields of view from the other set of duplicate samples.

### 2.8. Statistical Analysis

Statistical analysis of cell number, cell area and percentage pluripotency and their relationship to substrate topography and chemistry was evaluated using SPSS v22 by univariant analysis combined with a sidak post-hoc test, since there was interaction of the data, which made multivariant analysis inappropriate.

## 3. Results

### 3.1. Surface Design

**Figure 1:**
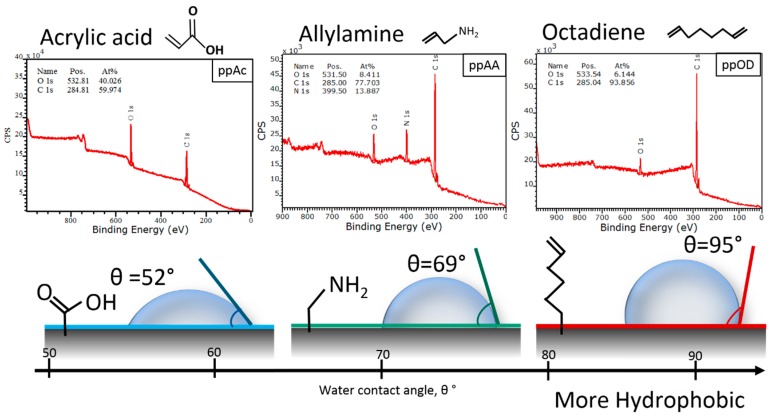
XPS survey spectra (**Top**) for plasma-deposited allylamine (ppAA), acrylic acid (ppAAc), and octadiene (ppOD), and (**Bottom**) corresponding water contact angles.

Representative XPS spectra of the different plasma polymer coatings together with the corresponding water contact angles are provided in Figure 1 [34]. The allylamine plasma polymer is the only coating containing nitrogen. The octadiene coating is primarily a hydrocarbon surface which nonetheless contain small amount of oxygen due to reactions between the reactive plasma-generated radicals and ambient oxygen species [35,36]. Finally the acrylic acid thin film is made of both carbon and oxygen, in comparable amount. This coating is significantly more hydrophilic than the others due to the presence of surface –COOH groups.

**Figure 2:**
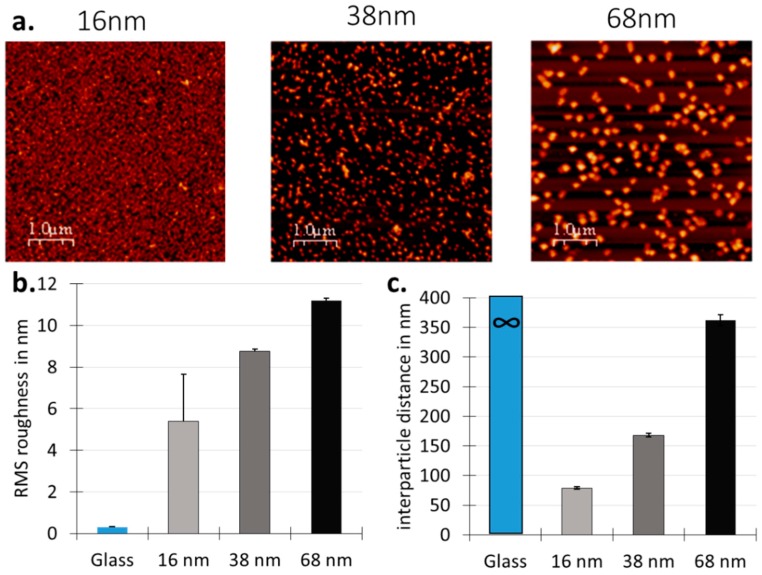
(**a**) Representative atomic force microscopy (AFM) images; (**b**) Root mean square (RMS) roughness and (**c**) Interparticle distance of surface with 16, 38 and 68 nm nanotopography.

Representative AFM images of the surface after immobilisation of 16, 38 and 68 nm gold nanoparticle are shown in Figure 2. Both the root mean square (RMS) roughness and interparticle distance increased as a function of nanoparticle size, in good agreement with previous studies [31].

### 3.2. Cell Behaviour

#### Cell Adhesion and Spreading

**Figure 3:**
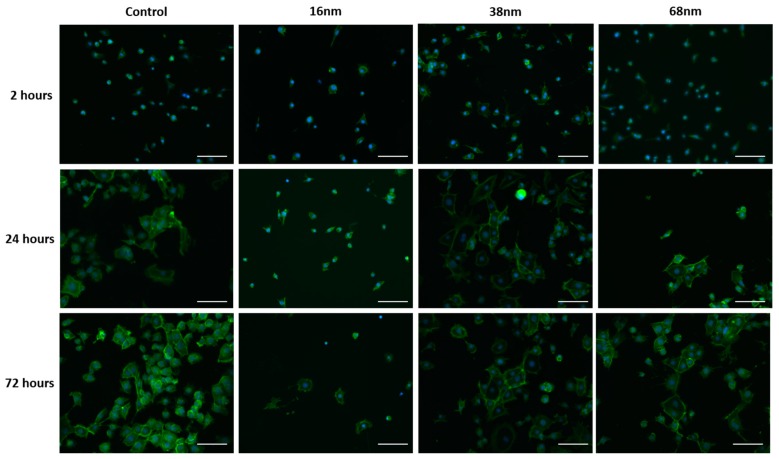
Cell adhesion and growth. Murine embryonic stem mES cells seeded on glass coverslips with and without treatment with 16, 38 and 68 nm particles precoated with fibronectin. Cells were cultured for 2, 24, and 72 h, fixed, and stained with Phalloidin actin stain (green) and DAPI nuclear stain (blue). Presented as representative images from each sample set. Scale bar = 100 mm.

Qualitative assessment of the effect of nanoparticle size over time on the cell morphology without further surface chemical modification (Figure 3) demonstrated that cells attached to all surfaces by 2 h, but that there was little spreading of the cells at this stage. By 24 h, the cells had spread more on the control surface and those with 38 nm and 68 nm particles than at 2 h, but those on the 16 nm surface maintained their less spread morphology. The same trend was observed after 72 h. 

**Figure 4:**
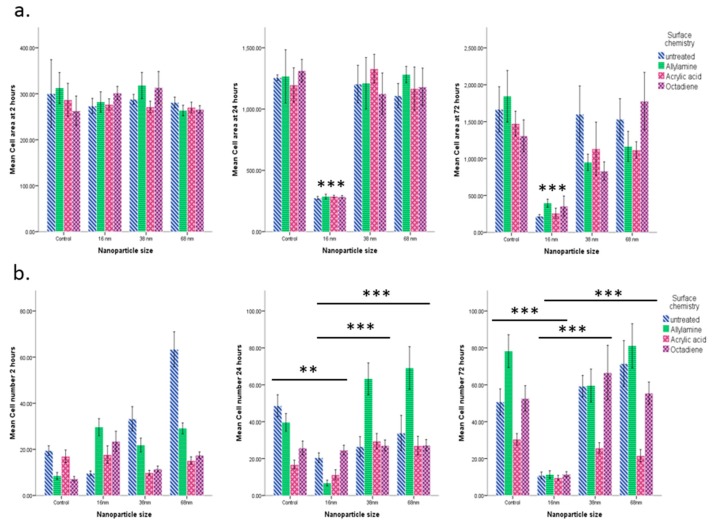
Effect of Nanotopography. mES cells seeded on glass coverslips with and without treatment with 16, 38, and 68 nm particles and subsequent surface chemical modification with allylamine, acrylic acid and octadiene, precoated with fibronectin. Cells were cultured for 2, 24, and 72 h, fixed, and stained with Phalloidin and DAPI. (**a**) Cell spreading: Cell area was calculated using Image J of all cells from five random fields of view on each sample. Presented as mean ± SD *n* = 5; (**b**) Cell proliferation: the number of cells were counted under phase contrast microscopy from eight random fields of view across duplicate samples for each condition. Presented as mean ± SD *n* = 8 (** = *p* < 0.01, *** = *p* < 0.001).

At 2 h there was no statistically significant difference in cell area as a function of nanoparticle size (Figure 4a). By 24 h there was a statistically significant reduction in cell area on those attached to the 16 nm particle surface in comparison with all other surfaces (*p* < 0.001). The same trend was observed at 72 h. 

In terms of cell number (Figure 4b) there was generally an increase in cell number between 2 h and 72 h, although this varied with relation to the specific surface properties. There was a significantly lower number of cells on the 16 nm particles surface than on the control (*p* < 0.001), 68 nm (*p* < 0.05), and 38 nm (*p* < 0.01) surfaces overall (comparing all time points and surface chemistry). By 24 h, the number of cells on the 16 nm particle surface was significantly lower than the control (*p* < 0.01), 38 nm (*p* < 0.001), and the 68 nm (*p* < 0.001) surfaces. By 72 h this trend had continued with there being significantly fewer cells attached to the 16 nm particle surface than all other surfaces (*p* < 0.001).

**Figure 5:**
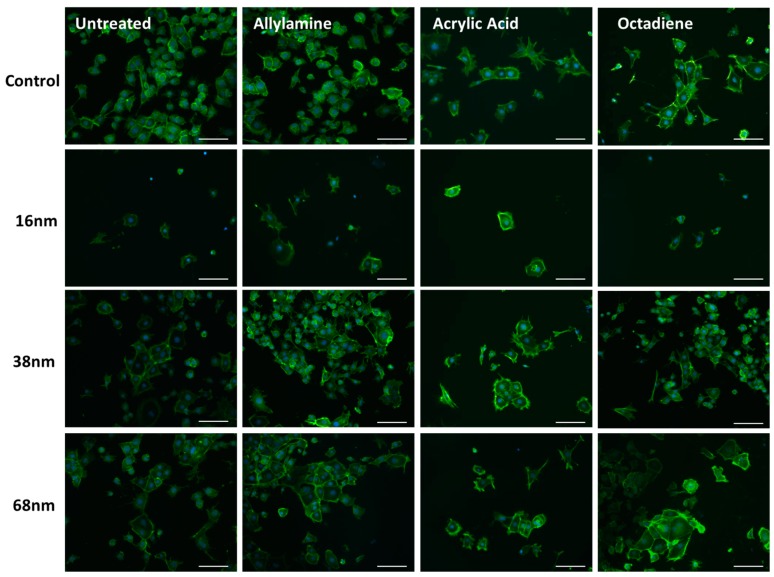
mES cells seeded on glass coverslips with and without treatment with 16, 38, and 68 nm particles and subsequent surface chemical modification with allylamine, acrylic acid and octadiene, precoated with fibronectin. Cells were cultured for 72 h, fixed and stained with Phalloidin and DAPI. Presented as representative images from each sample set. Scale bar = 100 mm.

When the influence of surface chemistry was imposed on top of the nanotopography, it was qualitatively observed that changing the surface chemistry of the 16 nm topography did not improve cell attachment, even by 72 h (Figure 5). Furthermore, it was clear that the acrylic acid surface chemistry caused a reduction in cell numbers on all levels of topography at this time point.

There was no statistically significant difference in cell area in relation to surface chemistry at any time point (Figure 4a). At 2 h there were significantly fewer cells on the acrylic acid and octadiene treated surfaces in comparison with the untreated surfaces (*p* < 0.001) (Figure 4b). By 24 h there were significantly fewer cells on the acrylic acid and octadiene treated surfaces than the allylamine-treated surfaces (*p* < 0.001 and *p* < 0.005, respectively). By 72 h there were significantly fewer cells on the acrylic acid treated surfaces than all other surfaces (*p* < 0.001) with no significant differences between the other three.

**Figure 6:**
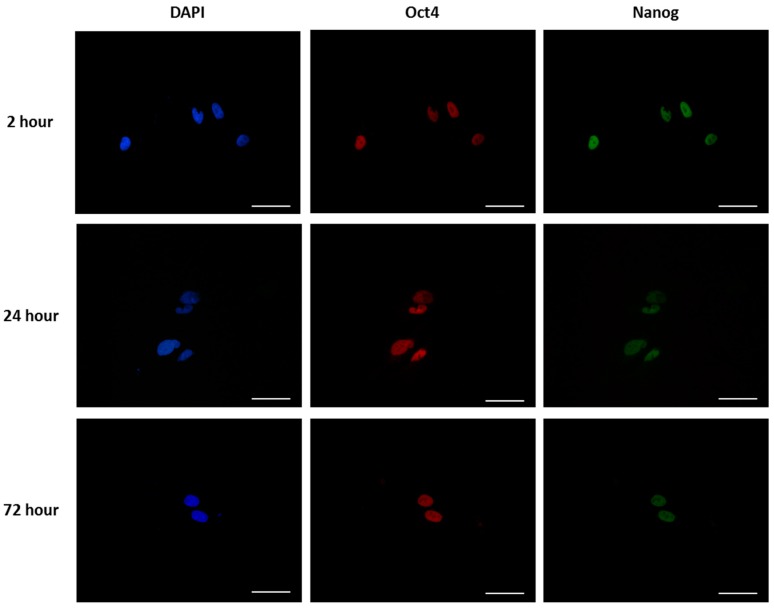
mES cells were grown on glass coverslips treated with 16 nm nanoparticles precoated with fibronectin for 2 h, 24 h, and 72 h. Cells were fixed, then stained with Oct4 and Nanog and counterstained with DAPI. Presented as representative images from each sample set Scale bar = 50 mm.

**Figure 7:**
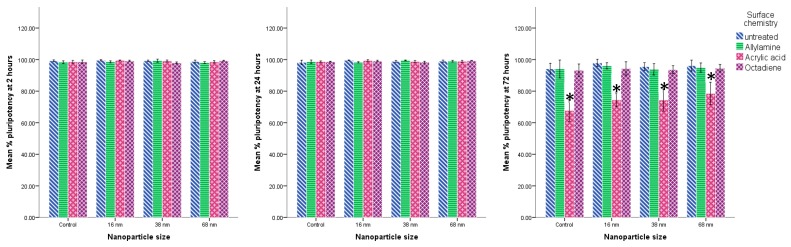
mES cells seeded on glass coverslips with and without treatment with 16, 38, and 68 nm particles and subsequent surface chemical modification with allylamine, acrylic acid and octadiene, precoated with fibronectin. Cells were cultured for 2, 24, and 72 h, fixed and stained with Oct4 and Nanog and counterstained with DAPI. The % of cells stained with Oct4/nanog to DAPI was counted in three random fields of view on each sample. Presented as mean ± SD *n* = 3 (* = *p* < 0.001).

Although there was a significant lack of proliferation and spreading of the cells on the 16 nm particle surfaces (equivalent images were obtained for other surface topographies, not shown) the cells that were attached maintained their markers of pluripotency at all three time points (Figure 6) and there was no statistically significant difference in the % of cells staining positive for pluripotent markers in relation to surface topography (Figure 7) at these time points.

**Figure 8:**
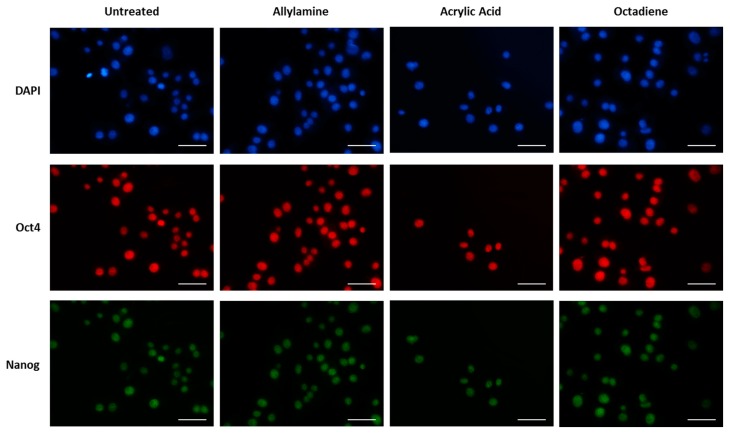
mES cells seeded on glass coverslips surface modified with allylamine, acrylic acid and octadiene, precoated with fibronectin. Cells were cultured for 72 h, fixed then stained with Oct4 and Nanog and counterstained with DAPI. Presented as representative images. Scale bar = 50 mm.

Surface chemical treatment with acrylic acid did reduce the ability of the attached cells to retain their pluripotency markers (Figure 8) at 72 h in comparison to all other surface chemical modifications and this reduction, although only an approximately 30% reduction, was statistically significant at this time point (*p* < 0.001) (Figure 7).

## 4. Discussion

In the current study, three different organic precursors and three different gold nanoparticle sizes were used to create surfaces with amine, carboxylic acid, or hydrocarbon chemistry, with and without 16, 38, and 68 nm nanotopography. These surfaces were then used to study the individual and combined effect of surface functionality and nanoroughness on mouse embryonic stem cells behaviour. We demonstrated that the topography and chemistry did not impact mES cells in the same way. Surface nanotopography influenced cell adhesion, spreading and proliferation, but had no significant effect on the ability of the cells to retain their markers of pluripotency. Chemistry, on the other hand, did not affect cell spreading significantly but played a role in proliferation and retention of pluripotency markers. More specifically, cell adhesion and proliferation was significantly decreased on surfaces with 16 nm nanoroughness, regardless of the overcoating chemistry, while it was comparable to the control surface for larger 38 and 68 nm nanoprotusions. One explanation of this observation may be in the manner in which proteins attach to these surfaces. Cell attachment to the artificial extracellular environment relies on the initial formation of a layer of adsorbed proteins [37], which occurs faster than cell settlement in culture conditions. Subsequently, cells surface receptors, such as integrins, recognise favourable active binding sites in the protein film formed on the culture substrate. The binding of integrin receptors to surface adsorbed biomolecules is a critical step in initiating an efficient cell adhesion process. In the present experiment, all substrates were initially incubated for 40 min in a fibronectin solution. Fibronectin is a large protein 15 nm in length and 9 nm in width. For the surfaces with 38 and 68 nm nanoparticles, the fibronectin molecule is small enough to bind to the surface around the particles and in between them, potentially providing surface binding sites for the cells. On the contrary, surfaces decorated with close-packed 16 nm particles (Figure 2a) may well be screening off the fibronectin, which is of comparable size, reducing the number of active binding sites for the cells. Thus, the surface nanoroughness size and associated steric effects are dictating the amount of the fibronectin that is able to bind to the substrates and thus influencing the cell attachment. This hypothesis is in good agreement with previous reports that demonstrated that the adsorption of protein was decreased or suppressed when the surface nanoroughness dimensions were comparable to that of the biomolecule [31,38]. Another way in which surface nanotopography may impact protein adsorption is by inducing conformational changes when the protein unfolds around the nanoparticles, as has been reported for fibrinogen [39,40]. In this situation, although fibronectin may be present, the active binding sites may no longer be available for the cells the bind to. Future work could further evaluate this by measuring the amount and conformation of the adsorbed fibronectin using quartz crystal microbalance with dissipation monitoring (QCM-D) [41,42]. The cells that did attach, however, maintained their pluripotency, suggesting that nanotopography was not a controlling factor in influencing the differentiation of these cells. Modification of the surface chemistry with polyacrylic acid, however, did hinder mSC proliferation and induced loss of pluripotency markers in a significant proportion of the cells. It has been reported that hydroxyl groups induce conformational changes in surface bound fibronectin [43]. It is therefore possible that the ppAAC surface modification which provides hydroxyl and carboxyl groups at the surface will interact with the adsorbing fibronectin to impact the conformation of fibronectin. If denaturation occurs, the mechanotransduction and the downstream cell behaviour may be altered in response to the surface chemical cue, and in this way would influence the differentiation potential of the cells. Future work could evaluate this further by analysing the presents and stability of focal adhesions between the cells and the underlying surface. Overall, our results could suggest that small nanotopography primarily reduced the level of fibronectin adsorbed on the surface, while the chemistry of ppAAc impacted their conformation, which respectively led to lower adhesion, spreading, and proliferation on the one hand, and lower self-renewal on the other. Our results are in good agreement with other studies indicating that the chemical and mechanical unfolding of proteins can lead to very different denaturation states [44,45]. Modifying culture substrates with extracellular matrix proteins to direct stem cell migration and differentiation is a vibrant field of research [46], mainly due to the fact that we still lack ways to control the orientation, conformation, and denaturation states of adsorbed proteins [47]. Using these results, we could design specific culture surfaces either to promote mES cell self-renewal to provide sufficient numbers of cells to be therapeutically relevant, or to drive differentiation of the cells for a specific application. 

## 5. Conclusions

In this work, plasma-generated substrates were used to investigate the effect of nanotopography and chemistry on mSC behaviour. It was demonstrated that nanotopography, when comparable in size to ECM cell adhesion proteins, hindered cell attachment, spreading and proliferation. Surface chemistry, however, did not impact cell attachment and spreading significantly, but carboxylic acid functionality did hinder cell proliferation and led to a reduction in their ability to retain pluripotency. The results highlight the distinct roles that topography and chemistry play in mSC fate in vitro.
